# Something Out of Nothing: A Rare Case of Pulmonary Renal Syndrome With Pauci-Immune Glomerulonephritis and Diffuse Alveolar Hemorrhage With Negative Serologies

**DOI:** 10.7759/cureus.18614

**Published:** 2021-10-08

**Authors:** Lucas Jiaxue Wang, Roberto Collazo-Maldonado

**Affiliations:** 1 Internal Medicine, Methodist Dallas Medical Center, Dallas, USA; 2 Nephrology, Methodist Dallas Medical Center, Dallas, USA

**Keywords:** gross hematuria, small vessel vasculitis, diffuse alveolar hemorrhage, anca negative, pauci-immune crescentic glomerulonephritis

## Abstract

Background: Pauci-immune crescentic glomerulonephritis (CrGN) is one of the most common etiologies of rapidly progressive glomerulonephritis. This condition presents with crescentic glomerulonephritis with little or no immunoglobulin staining and negative serological workup aside from a positive antineutrophil cytoplasmic autoantibody (ANCA). Typically, patients with pauci-immune CrGN have an underlying systemic small vessel vasculitis, but in rare cases, it presents without any known vasculitis or ANCA. Pauci-immune ANCA negative CrGN is often strictly isolated to the kidneys. In this case, we present a patient with ANCA negative, pauci-immune CrGN with severe diffuse alveolar hemorrhage.

Case Presentation: A 66-year-old Hispanic woman with a past medical history of controlled hypertension presented with fatigue and dysphagia. On admission, her vital signs were significant for hypoxia on room air, and her physical exam was remarkable for crackles bilaterally. The initial laboratory results revealed anemia (hemoglobin 5.2 g/dL), hyperkalemia (6.3 mmol/L), elevated creatinine (4.50 mg/dL, with a baseline of 0.9mg/dL). Urinalysis showed moderate blood and urine protein (200 mg/dL). Urine microscopic examination showed 25-50 RBCs seen/high power field.

The patient was admitted to ICU due to hypoxia, a computed tomography scan of the chest/abdomen/pelvis was obtained and revealed multifocal pulmonary consolidations. A blood transfusion was ordered. The patient began to have hemoptysis and subsequent bronchoscopy showed diffuse alveolar hemorrhage. ICU team proceeded to intubate her as the hemorrhage continued to worsen. Further workup revealed a positive anti-nuclear antibodies (ANA) of 1:40, but otherwise negative serologies including myeloperoxidase (MPO)-ANCA, glomerular basement membrane antibody, and anti-double stranded DNA. Kidney biopsy showed necrotizing glomerulonephritis with crescents and negative immunofluorescence. She was diagnosed with pauci-immune ANCA-negative vasculitis with associated diffuse alveolar hemorrhage and nephritis based on these results and was started on pulse-dose steroids. The patient was started on intravenous (IV) high-dose cyclophosphamide, which helped improved the overall clinical condition significantly. After creatinine began trending down and urine output improved, the patient was discharged on a regimen of daily oral cyclophosphamide and steroid taper. Patient oxygen requirements decreased and she was sent home with supplemental oxygen while requiring 3L/min of oxygen.

Conclusion: Pauci-immune and ANCA-negative glomerulonephritis with concurrent diffuse alveolar hemorrhage is exceptionally rare. In this situation, medical management relied on clinical evidence from similar populations in the use of steroids and cyclophosphamide. This case report aims to shed more light on the clinical progression and management of this condition. Here we present a case of pulmonary-renal syndrome with biopsy-proven glomerulonephritis but without ANCA positive serologies.

## Introduction

Pauci-immune crescentic glomerulonephritis (CrGN) is one of the three most common etiologies of rapidly progressive glomerulonephritis, a subset of small vessel vasculitis (SV) [1,2]. The characteristic feature of this condition is a crescentic glomerulonephritis with little or no immunoglobulin staining and negative serological workup aside from a positive p-ANCA or PR3-ANCA [3]. Typically, patients with pauci-immune CrGN have an underlying systemic small vessel vasculitis, but in rare cases, it is not associated with any known systemic vasculitis or antineutrophil cytoplasmic autoantibody (ANCA). Moreover, pauci-immune ANCA negative CrGN is often strictly isolated to the kidneys. Various reports have shown that the key difference in presentation between ANCA positive and ANCA negative pauci-immune CrGN is the severity and frequency of respiratory symptoms associated with this condition, with ANCA [4,5]. In this case, we present a patient with ANCA-negative, pauci-immune CrGN with severe diffuse alveolar hemorrhage (DAH).

## Case presentation

A 66-year-old Hispanic woman with a past medical history of hypertension presented to the emergency department with worsening shortness of breath and hemoptysis for the last 24 hours was admitted to our hospital in February 2021. She went to the emergency department for worsening hemoptysis and fatigue. Her background medical history consisted of hypertension controlled by amlodipine alone. She had no known history of autoimmune or connective tissue disease.

She was previously hospitalized for severe anemia (hemoglobin of 5.2 mg/dL) without hemoptysis at an outside hospital requiring five units of blood transfusion. Her initial creatinine outside hospital was 4.5 mg/dL, with a previous baseline of 0.9 mg/dL, and began trending down prior to discharge. Of note, the patient had a chest X-ray that showed possible lung cysts suggestive of emphysema which were attributed to secondhand smoke. She was discharged but unfortunately returned to the emergency department the next day for worsening shortness of breath and fatigue.

**Figure 1:**
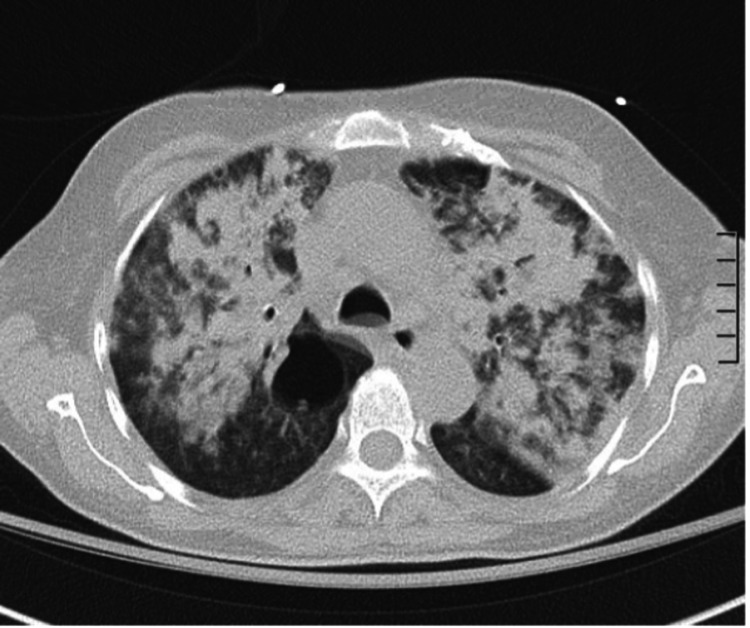
Computed tomography scan of the chest showed diffuse airspace opacities within the lungs bilaterally

**Figure 2:**
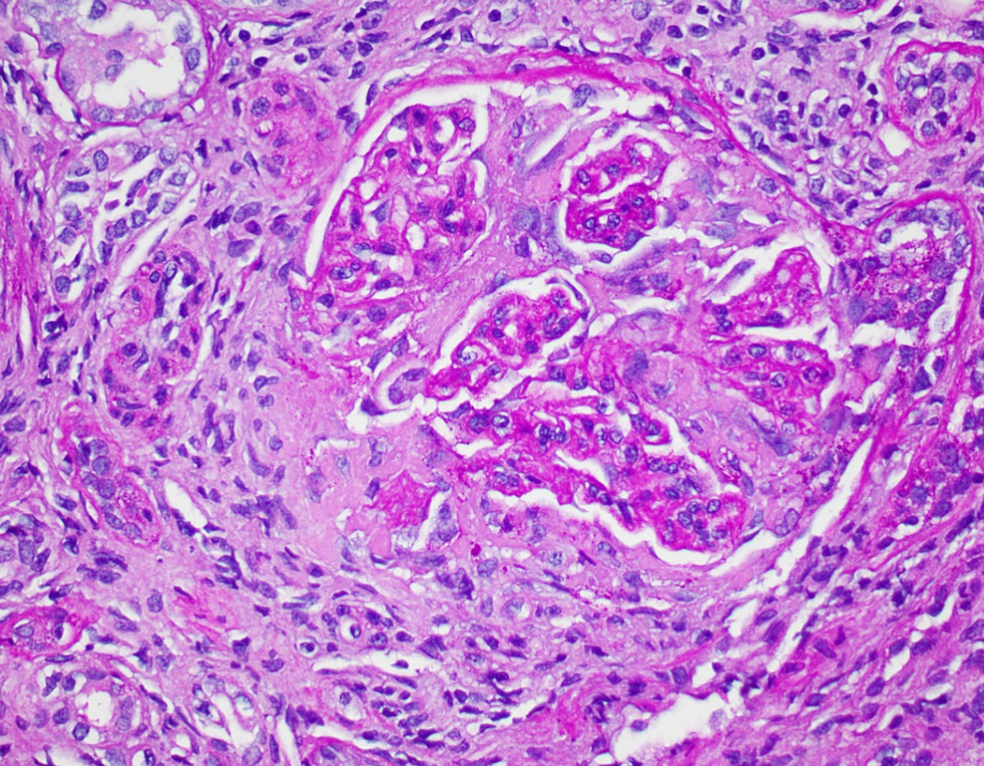
Kidney biopsy showed glomerular scarring (H&E staining)

On arrival, the patient reported worsening hemoptysis alongside her previous symptoms. She denied nausea, vomiting, cough, oliguria, or diarrhea. Her vital signs were normal, and her physical examination was only remarkable for coarse crackles on both lungs. Shortly after her arrival to our emergency department, the patient began requiring 8L of oxygen. Initial lab results (Table 1) revealed low hemoglobin on arrival with concomitant elevated creatinine and NT-proBNP (N-terminal pro b-type natriuretic peptide). Urinalysis showed hematuria (3+) and proteinuria (2+). She was admitted for pulmonary-renal syndrome with a concern for autoimmune vasculitis in the setting of an acute kidney injury and hemoptysis. A computer tomography (CT) scan of the chest (Figure 1) was ordered due to concern of pulmonary involvement. The image showed worsening diffuse airspace opacities within the lungs bilaterally. Bronchoscopy showed diffuse alveolar hemorrhage. The patient required mechanical ventilation shortly after the procedure. Patient’s creatinine and hemoptysis improved significantly after treatment with daily 60mg IV methylprednisolone and 1000mg/m^2 ^of onetime IV cyclophosphamide. A kidney biopsy was done following resolution of her diffuse alveolar hemorrhage (Figure 2). The results were pauci-immune necrotizing glomerulonephritis with 10% cellular crescents, segmental glomerular scarring, and advanced interstitial fibrosis and tubular atrophy (70%) with associated acute tubular injury. Also of note, ANCA titers were repeated twice and they were negative on both occasions. Her creatinine levels (Table 2) improved with stable urine output and patient was discharged on home oxygen.

**Table 1:** The patient’s initial workup ANA: antinuclear antibody; ANCA: antineutrophil cytoplasmic antibodies; CRP: C-reactive protein; ESR: erythrocyte sedimentation rate; GBM: glomerular basement membrane; HBsAg: hepatitis B surface antigen; INR: international normalized ratio; HCV: hepatitis C virus; LDH: lactate dehydrogenase; SPEP: serum protein electrophoresis; UPEP: urine protein electrophoresis; PT: prothrombin time; aPTT: activated partial thromboplastin time; MPO: myeloperoxidase; PR3: proteinase 3; C3, C4: complement component 3 and 4.

Test Name	Reference Range	Lab Value
Hematology		
Hemoglobin (mg/dL)	12.0 – 16.0	6.5
Platelet Count (x10^3^/uL)	130 – 400	211
LDH (U/L)	313 – 618	413
D-Dimer, Quant (ug/mL)	0.00 – 0.50	5.4
Fibrinogen (mg/dL)	214 – 481	382
aPTT (seconds)	23 – 37	21
PT	11.3 – 14.7	15.2
INR	0.9 – 1.2	1.2
Lactic Acid (mmol/L)	0.40 – 2.00	1.50
Rheumatology Panel		
ANA titer	Negative at 1:40	1:40
Anti-DNA antibody (IU)	<= 20.000	5.269
GBM Ab IgG (AU/mL)	0 – 19	0
MPO-ANCA (AU/mL)	0 – 19	0
PR3-ANCA (AU/mL)	0 – 19	0
Anti-Phospholipase A2	>19 RU/mL	<2 RU/mL
Kappa Free Light Chain (mg/L)	3.30 – 19.40	191.40
Lambda Free Light Chain (mg/L)	5.71 – 26.30	161.94
Kappa/Lambda Ratio	0.26 – 1.65	1.18
SPEP	Negative	Negative
UPEP	Negative	Negative
Inflammatory Markers		
Procalcitonin (ng/mL)	<0.25	1.33
CRP (mg/L)	<=10	233
ESR (mm)	0-20	43
C3 (mg/dL)	88 – 165	76
C4 (mg/dL)	14 – 44	42
Infectious workup		
HCV Ab		Negative
HBsAg		Negative
Blood Culture		No growth at five days

**Table 2:** Kidney function during patient’s length of stay Prednisone was applied on Day 5. Cyclophosphamide was applied on Day 7.

Test Name	Reference Range	Day 1	Day 2	Day 3	Day 4	Day 5	Day 6	Day 7	Day 8	Day 9	Day 10	Day 11	Day 12
Creatinine (mg/dL)	0.7 – 1.3	3.09	3.30	3.50	3.30	2.80	3.10	2.80	2.30	2.50	2.30	2.10	1.90

## Discussion

Our patient was initially admitted with anemia, DAH, and a recent history of acute kidney injury. Initial workup for malignancy and infection was negative. The patient was diagnosed with pauci-immune ANCA-negative CrGN based on kidney biopsy and negative serologies including ANCA. Severe pulmonary issue such as DAH, to our knowledge, has never been observed with pauci-immune ANCA-negative CrGN.

**Figure 3:**
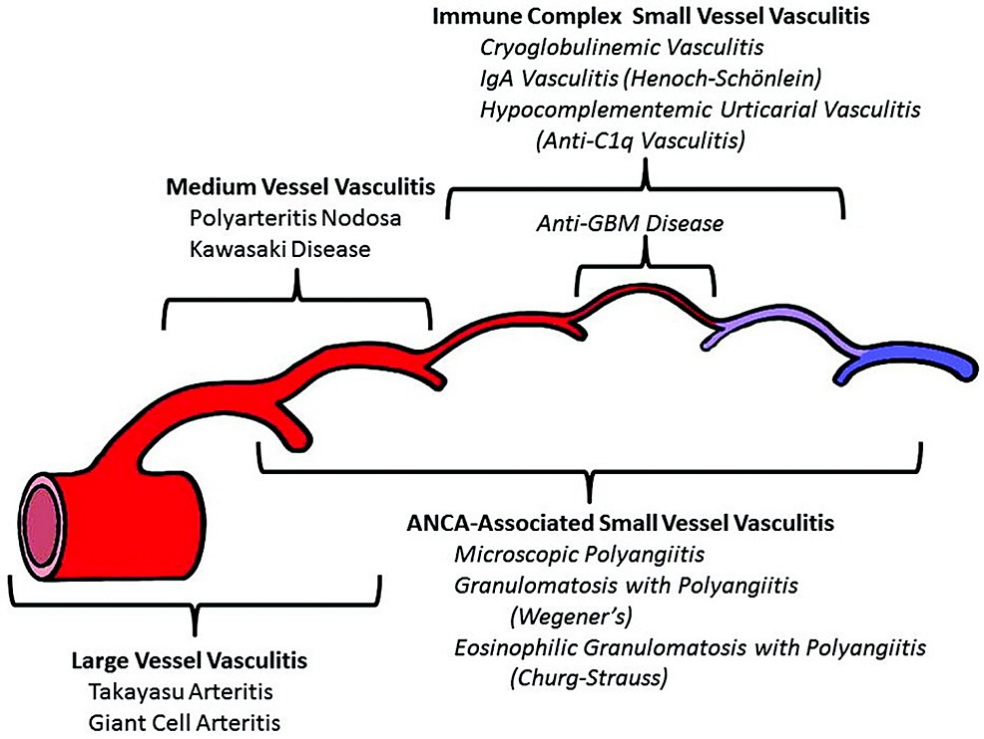
2021 Revised International Chapel Hill Consensus Conference Nomenclature of Vasculitides ANCA: antineutrophil cytoplasmic antibodies; GBM: glomerular basement membrane

Pauci-immune ANCA-negative CrGN with DAH is a rare combination of conditions that presented in this patient. When comparing to other types of vasculitis, pauci-immune ANCA-negative CrGN predominantly affects a wide range of small arteries and veins [1] (Figure 3). Classically, pauci-immune CrGN presents as necrotizing and crescentic glomerulonephritis with little or no glomerular staining for Ig by immunofluorescence microscopy or complements [2]. Ninety percent of patients with this condition present with an ANCA-positive biopsy [2,3]. Previous studies have shown that pauci-immune ANCA-negative CrGN patients almost always have renal manifestations. Patients with pauci-immune ANCA-negative CrGN have kidney biopsies that show 50-100% crescentic glomerulonephritis. Fibrinoid necrosis, however, was only seen in 0-3% of cases. Compared with ANCA-positive pauci-immune CrGN, pauci-immune ANCA-negative CrGN had a significantly increased number of abnormal glomeruli and a higher percentage of cellular crescent formation [4]. Findings in kidney biopsy that support pauci-immune ANCA-negative CrGN diagnosis include focal crescentic glomerulonephritis

In addition, pauci-immune ANCA-negative CrGN is often shown to have fewer and milder extrarenal symptoms than their ANCA-positive counterparts [4]. Recent studies have reported around 10% of pauci-immune ANCA-negative CrGNA can have pulmonary manifestation such as hemoptysis or pulmonary nodules, but never has a case of DAH been reported [5]. It is largely believed that ANCA plays an important role in pauci-immune CrGN. The fact that this patient presented with life-threatening DAH is an unusual phenomenon in this condition. Despite the decreased number of extrarenal manifestations, pauci-immune ANCA-negative CrGN is still associated with higher mortality rates than ANCA positive pauci-immune CrGN, especially in the elderly population [6].

Treatment for ANCA vasculitis has progressed steadily over the last few decades. Landmark trials continue to guide treatment plans for a huge variety of small vessel vasculitis (Table 4). Medical management for this patient followed the principles set by the new PEXIVAS [7] trial despite it being restricted to ANCA-associated vasculitis. Previously, plasmapheresis was the choice of treatment; but this new trial shows that in the setting of diffuse alveolar hemorrhage there is no benefit for plasmapheresis in terms of mortality and progression to end-stage kidney disease. Standard dose glucocorticoids followed by cyclophosphamide treatment were started instead and patient condition stabilized afterward.

**Table 3:** Landmark trials on treatment for ANCA-associated vasculitis ANCA: antineutrophil cytoplasmic antibodies; ESKD: end-stage kidney disease

Trial	Year	Description
PEX	1990	Plasma exchange provided additional benefit to dialysis-dependent patients who had focal necrotizing glomerulonephritis without anti-GBM antibodies, but 12 month follow-up showed this difference was less pronounced [8].
CYCAZAREM	2003	Induction cyclophosphamide can safely be transitioned to maintenance azathioprine in vasculitis associated with antineutrophil cytoplasmic antibodies [9].
MEPEX	2007	PLEX showed reduction in risk of progression to ESRD in patients with serum creatinine >5.7 mg/dL when compared to high dose steroids for those with granulomatosis with polyangiitis and microscopic polyangiitis, but longer term follow-up showed that the difference diminished after 12 months for ESKD and mortality [10].
WEGENT	2008	Methotrexate and azathioprine are similar alternatives for maintenance therapy in patients with granulomatosis with polyangiitis and microscopic polyangiitis [11].
CYCLOPS	2009	Neither oral or intravenous cyclophosphamide were shown to be more likely to induce remission and at a faster rate [12].
IMPROVE	2010	Among patients with ANCA associated vasculitis, mycophenolate was shown to have higher rates of disease remission than azathioprine [13].
RAVE	2010	Rituximab therapy was shown to be not inferior to daily cyclophosphamide treatment for induction of remission in severe ANCA associated vasculitis and may be superior in relapsing disease [14].
Rituxivas	2010	A rituximab based regimen was not superior to standard intravenous cyclophosphamide for severe ANCA associated vasculitis [15].
MAINRITSAN	2014	More patients with ANCA-associated vasculitides had sustained remission at month 28 with rituximab than with azathioprine [16].
PEXIVAS	2020	Among patients with severe ANCA-associated vasculitis, the use of plasma exchange did not reduce the incidence of death or ESKD. A reduced-dose regimen of glucocorticoids was non-inferior to a standard-dose regimen with respect to death or ESKD [7].

All in all, patients who present with ANCA-negative vasculitis with pauci-immune CrGN typically do not have significant systemic involvement particularly when it relates to the pulmonary system. Renal lesions are characterized by necrotizing and crescentic glomerulonephritis. Immunosuppressive drugs in the form of corticosteroids, cyclophosphamide and rituximab can bring about significant improvement in the acute setting and overall prognosis. Further research is needed to better understand the pathogenesis of these systemic vasculitides to improve diagnostic markers and treatment modalities.

## Conclusions

Pauci-immune ANCA-negative CrGN is a rare subset of pauci-immune CrGN occurring in only 10% of all cases. Even rarer is this causing a life-threatening DAH requiring prompt intubation. Prompt recognition and intensive treatment with steroids and cyclophosphamide treatment were critical in mitigating unfavorable outcomes.
